# Right heart catheterization blended training program: quality improvement and educational outcomes

**DOI:** 10.1093/atsscholar/aapag022

**Published:** 2026-04-28

**Authors:** Mary Jo S Farmer, Aleezay Asghar

**Affiliations:** Division of Pulmonary, Critical Care, and Sleep Medicine, Mass General Brigham, Salem, MA, United States; Division of Pulmonary, Critical Care, Sleep, and Allergy, University of Illinois Chicago, Chicago, IL, United States

**Keywords:** pulmonary arterial hypertension, pulmonary hypertension, education, cardiac output, hemodynamics, thermodilution, Fick, pulmonary arterial wedge pressure

## Abstract

**Background:**

Accurate measurement and reporting of pulmonary arterial wedge pressure (PAWP) and cardiac output (CO) are essential for diagnosing and managing pulmonary hypertension and pulmonary arterial hypertension (PAH). Errors in these parameters can lead to misclassification and inappropriate treatment. Recent updates from the 2023 European Respiratory Society/European Society of Cardiology guidelines and the 2024 Seventh World Symposium on Pulmonary Hypertension emphasize the need for standardized right heart catheterization (RHC) practices.

**Objective:**

To identify knowledge gaps in RHC practices among interprofessional team members and implement a targeted blended learning program developed for pulmonary critical care and cardiology fellows, with a focus on accurate acquisition and interpretation of PAWP and CO.

**Methods:**

Using the SQUIRE framework, we conducted a baseline survey assessing RHC practices and knowledge among pulmonary critical care and cardiology attending physicians and fellows and critical care nurses at an academic medical center. Respondents were actively involved in RHC procedures across the cardiac catheterization laboratory, medical intensive care unit, and cardiac care unit. Survey items with < 70% correct response were used to guide development of a 120-minute blended learning program incorporating lectures, video instruction, and hands-on-simulation. Pre- and posttraining assessments were conducted.

**Results:**

Of 141 invited participants, 85 responded to the survey (60.3% response rate). Baseline data revealed knowledge gaps and therefore opportunities for learning in CO measurement methods and PAWP interpretation. Assessment postblended learning program demonstrated knowledge improvements in key areas including identification of indirect Fick (iFick) as the most common CO method, confirmation of zero-reference level by the proceduralist-nurse team, acquisition of PAWP, and obtaining at least 3 measurements of CO via thermodilution. However, correct identification of end-expiratory PAWP declined, highlighting the need for greater emphasis on this skill in future training sessions.

**Conclusion:**

This is the first reported interprofessional training initiative to target CO and PAWP measurement accuracy in RHC. This study identified key knowledge gaps and therefore opportunities for learning. Knowledge improvements measured after the educational intervention support the need for ongoing, structured, and interprofessional training to enhance procedural competency and interpretation of RHC hemodynamic data to optimize pulmonary hypertension and PAH care.

## Introduction

Pulmonary hypertension is a hemodynamic condition defined by a mean pulmonary artery pressure greater than 20 mmHg, as confirmed by right heart catheterization (RHC).^1-3^ While echocardiography can estimate pulmonary artery systolic pressure using the Bernoulli equation supplemented by assessments of right ventricular (RV) size, function, wall thickness, and interventricular septal configuration,^2^ RHC remains the gold standard for definitive diagnosis.

Accurate hemodynamic measurements are essential for proper classification of pulmonary hypertension and for guiding therapeutic decisions, particularly regarding appropriate use of pulmonary vasodilators. Key parameters obtained during RHC include pulmonary arterial wedge pressure (PAWP), pulmonary vascular resistance (PVR), and cardiac output (CO), which are central to current classification schemes.^1^^,^^3^ Inaccuracies in PAWP or CO can result in misclassification of pulmonary hypertension subtype, inappropriate selection of vasodilator therapy (eg, intravenous vs oral/inhaled agents), unnecessary repeat catheterizations, flawed preoperative risk assessments, and misapplication of clinical trial eligibility criteria.^4^^,^^5^ Pulmonary arterial wedge pressure is especially critical for distinguishing precapillary pulmonary hypertension (group 1 pulmonary arterial hypertension) from postcapillary pulmonary hypertension (group 2), while CO is required to calculate PVR, which differentiates isolated postcapillary pulmonary hypertension from combined pre- and postcapillary forms (Table S1).

Although direct Fick (dFick) is considered the gold standard for measuring CO, its use is limited in clinical practice due to the technical challenges of measuring oxygen consumption bedside. Thermodilution (TD) is preferred in most cases, except when intracardiac shunts are present.^6^^,^^7^ Indirect Fick (iFick), while commonly reported, relies on estimated oxygen uptake and is not recommended as a standalone method.^1^ Consequently, current guidelines recommend obtaining both TD and iFick measurements during RHC for pulmonary hypertension.^1^^,^^3^

Multiple studies highlight limitations in CO measurement techniques. Indirect Fick tends to underestimate CO compared to TD, potentially leading to misclassification of pulmonary hypertension severity.^8-10^ When discrepancies arise, TD is generally favored.^11^ However, even TD may underestimate CO relative to dFick, affecting risk stratification in a significant proportion of cases.^12^

Concerns regarding the accuracy and interpretation of hemodynamic data have been longstanding. A seminal study^13^ administered a 31-item multiple-choice examination to 496 physicians across 13 institutions in the United States and Canada to assess knowledge of RHC use and data interpretation. The mean test score was 67%, with a wide range (19%-100%), prompting calls for documented competency standards. A subsequent study^14^ evaluated 216 nurses preregistered for a hemodynamics workshop revealing a mean score of 48.5%. Factors such as catheter manipulation responsibilities, frequency of use, and years of critical care experience were significantly associated with performance. These findings underscored the need to reevaluate educational practices and consider credentialing policies. Further evidence of variability in interpretation was demonstrated in a study comparing PAWP measurements recorded by critical care nurses and interpreted by the chiefs of critical care and cardiology.^15^ The greatest discrepancies occurred in tracings with pronounced respiratory variation, highlighting a key area for targeted education. More recently, a review of 182 PAWP tracings at a large academic center identified alternative wedge pressures—defined as a pressure during balloon deflation ≥ 3 mmHg lower than the reported value—in 14.3% of cases. In 38.5% of these, the alternative wedge pressure reclassified the pulmonary hypertension etiology from postcapillary to precapillary.^16^

These studies included cardiologists, pulmonologists, intensivists, and nurses, emphasizing that accurate placement of the RHC and interpretation of hemodynamic data require effective interprofessional collaboration and teamwork. Interprofessional collaboration refers to multiple professions working together toward shared goals,^17^ while teamwork in health care is defined by shared identity, clarity of roles, interdependence, integration, and collective responsibility.^18^ Ineffective teamwork and team communication are associated with increased patient harm and sentinel events.^19-22^ There is growing recognition of the need for interprofessional approaches to health care delivery, particularly in the context of increasing complexity and the shift to precision medicine.^23^ While diverse teams bring broader expertise and resources to clinical challenges, effective collaboration requires more than assembling individuals from different disciplines. High-performing teams demonstrate frequent interaction, clarity of roles and goals, inclusive participation, psychological safety, and commitment to a shared vision.^24^^,^^25^

### Opportunity for knowledge improvement

Recent updates to RHC standards for pulmonary hypertension—published in the 2023 European Respiratory Society/European Society of Cardiology guidelines and reaffirmed during the 2024 Seventh World Symposium on Pulmonary Hypertension—emphasize the importance of standardized procedural and reporting practices.^1^^,^^3^ Expert consensus further supports inclusion of pressure waveforms from all key anatomical positions, as well as pulmonary artery wedge oxygen saturation (PAWO_2_), particularly when the interpreting clinician is not the proceduralist.^26-28^

At the time of this study, institutional RHC practices had not yet fully aligned with these updated recommendations. Many patients referred to pulmonary hypertension clinic presented with hemodynamic data from RHCs performed internally. Cardiac output was typically reported using iFick, never dFick, and TD CO was infrequently documented. While pressure waveform snapshots from the right atrium, RV, pulmonary artery, and pulmonary artery wedge positions were routinely available in the electronic medical record for RHCs performed in the cardiac catheterization laboratory, such documentation was often absent for RHCs conducted elsewhere. Accordingly, our project pursued 2 primary objectives. First, we conducted a comprehensive survey of current practices among the interprofessional team responsible for acquiring and reporting RHC hemodynamic data at our academic medical center. Second, recognizing that optimal RHC performance requires anatomical knowledge, procedural skill, clinical reasoning, and effective interprofessional collaboration and teamwork, we analyzed survey findings to identify knowledge gaps. These knowledge gaps translated to opportunities for learning and informed the development of a targeted educational intervention, delivered via a blended learning platform and codesigned with interprofessional team members.

The educational goals of the intervention were to foster conceptual understanding of RHC in the context of pulmonary hypertension, promote clinical reasoning for appropriate indications, build technical proficiency through simulation, and enhance interprofessional collaboration. Emphasis was placed on accurate acquisition and interpretation of PAWP and CO, given their central role in the diagnosis and management of pulmonary hypertension.

## Methods

This single center study was conducted at a tertiary care academic medical center. The project was reviewed and deemed exempt by the institutional review board (IRBNetID 2005507-2).

### Survey design and implementation

The initial phase of the study followed the Revised Standards for Quality Improvement Reporting Excellence (SQUIRE 2.0) guidelines.^29^ A semiquantitative survey (Supplement) was developed using REDcap^30^ by the study team comprising an internal medicine resident, a research coordinator, and a pulmonary critical care medicine (PCCM) attending physician. The survey aimed to assess practice and knowledge related to standard RHC procedural technique.^31-33^ Survey questions queried establishing the zero-reference level (ie, phlebostatic axis) in the supine position;^34^ measuring PAWP during the respiratory cycle; identifying methods for measuring CO; confirming PAWP position using PAWO_2_ saturation; documenting hemodynamic waveforms; identifying preferred insertion site of the introducer and right heart catheter; and using fluid challenge, vasoreactivity testing, and esophageal pressure measurement.

The survey underwent 5 iterative rounds of revision, incorporating feedback from a statistical consultant. Three subject matter experts (a PCCM attending, a senior critical care nurse, and a third-year PCCM fellow) piloted the survey. The final version included 25 items in multiple-choice (best answer), Likert scale, and open-ended formats. The survey was programmed in REDcap by the research coordinator and finalized by the principal investigator.

Survey invitations were distributed via automated REDCap emails over a 5-week period, with weekly reminders. Eligible participants included attending physicians and fellows in cardiology and PCCM, as well as critical care nurses. All participants were actively involved in RHC procedures performed in the cardiac catheterization laboratory, medical intensive care unit, or cardiac care unit.

### Performance threshold

A performance threshold of 70% correct responses was applied to survey items to identify areas of adequate vs insufficient knowledge. This cutoff is consistent with standards commonly used in medical education and competency-based assessment, where a score of 70% is considered indicative of minimum acceptable performance.^35^ Similar benchmarks are employed in certification examinations, continuing medical education posttests, and mastery learning frameworks to ensure learners demonstrate sufficient understanding before advancing to higher-stakes tasks.^35^ Survey items falling below this threshold were interpreted as indicators of knowledge gaps, reflecting the critical nature of clinical decision making in pulmonary hypertension.

### Educational intervention

**Figure 1:**
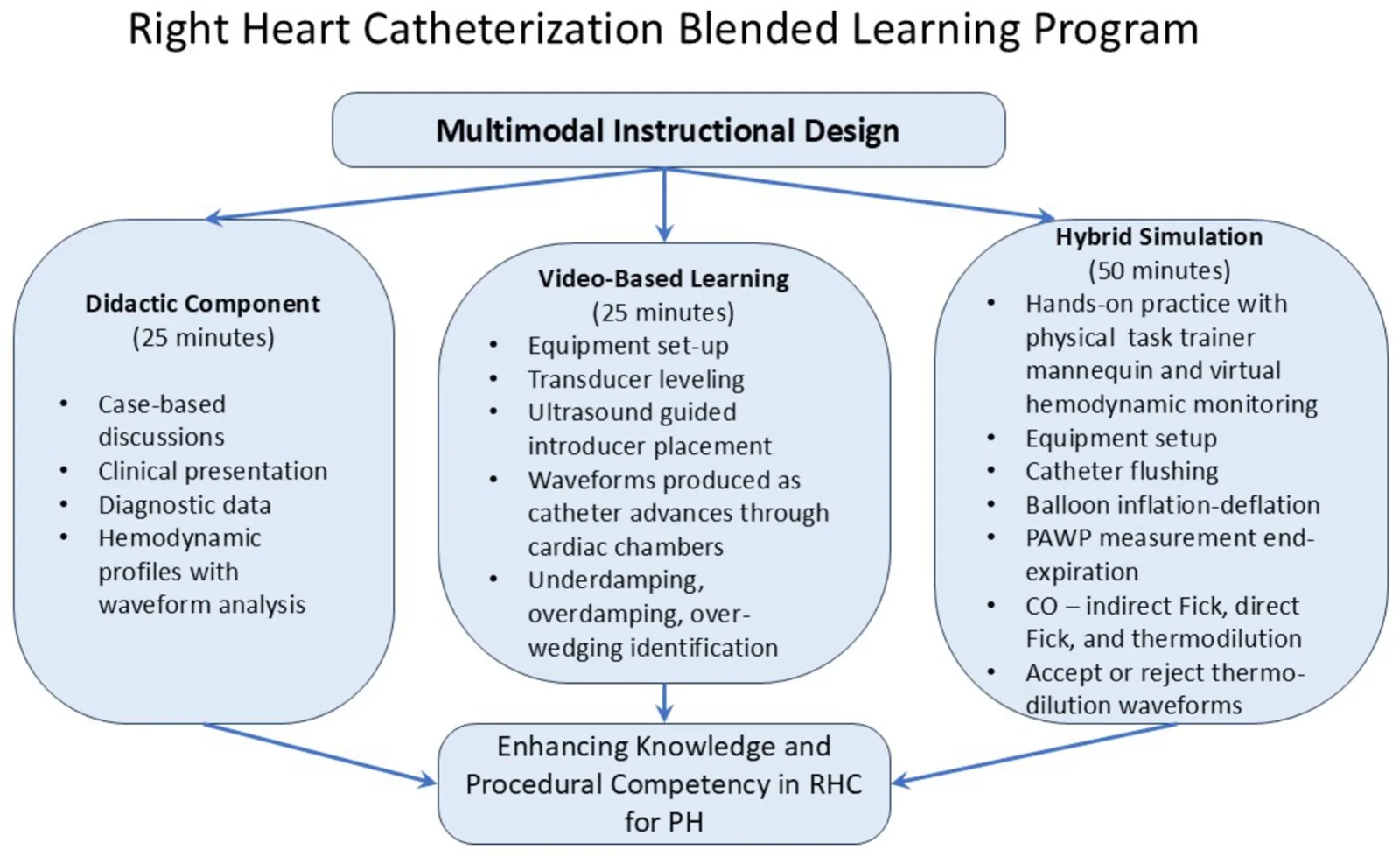
Right heart catheterization in pulmonary hypertension blended learning program.

The second phase of the project consisted of a 120-minute blended learning program^36^ aimed at the RHC team that used a multimodal instructional design to enhance knowledge and procedural competency in RHC for pulmonary arterial hypertension (PAH) **(**Figure 1). The curriculum comprised didactic instruction (25 minutes), video-based learning (25 minutes), and hybrid simulation (50 minutes) and was explicitly aligned with core competency-based standards established by the Accreditation Council for Graduate Medical Education (ACGME) for PCCM and cardiology fellowship training. Under recently revised ACGME requirements, PCCM fellows are expected to demonstrate knowledge of the indications, contradictions, and complications of RHC, while cardiology fellows must demonstrate competence in safely performing the procedure, interpreting hemodynamic data, and understanding procedural indications and risks.^37^ The alignment between educational content and learning objectives is summarized in Table 1.

**Table 1:** Alignment of educational content and learning objectives.

Educational content	Corresponding learning objectives
**Indications for RHC in pulmonary hypertension**	Identify clinical signs and symptoms prompting RHC for pulmonary hypertension
**Equipment setup and transducer leveling**	Recognize procedural complications and limitations
**Catheter placement and waveform identification**	Recognize procedural complications and limitations
**Hemodynamic interpretation**	Interpret hemodynamic data including CO, PAWP, and PVR; integrate RHC findings into pulmonary hypertension/PAH management
**Case-based discussions and instructional videos**	Understand the roles of interprofessional team members in RHC; participate in team-based interpretation
**Hemodynamic profiles of pre- and postcapillary pulmonary hypertension**	Interpret hemodynamic data including CO, PAWP, and PVR
**Methods for CO measurement (TD, dFick, iFick)**	Interpret hemodynamic data including CO, PAWP, and PVR
**Troubleshooting waveform artifacts and catheter positioning**	Recognize procedural complications and limitations

Abbreviations: CO, cardiac output; dFick, direct Fick; iFick, indirect Fick; PAH, pulmonary arterial hypertension; PAWP, pulmonary arterial wedge pressure; PVR, pulmonary vascular resistance; RHC, right heart catheterization; TD, thermodilution.

#### Didactic component

The didactic component was led by a PCCM attending and employed case-based instruction integrating clinical presentation with key diagnostic data, including N-terminal pro-B-type natriuretic peptide, pulmonary function testing, echocardiography, 6-minute walk testing, rest and exercise heart rate and oxygen saturation, chest computed tomography, ventilation-perfusion imaging, and RHC hemodynamics with waveform analysis. Instruction emphasized differentiation of pre- and postcapillary pulmonary hypertension (Table S1) and reviewed CO measurement techniques using TD, dFick, and iFick methods (Table S2). Video-based modules (https://pdf4pro.com/view/cath-lab-essentials-basic-hemodynamics-for-the-cath-lab-61203c.html) demonstrated ultrasound-guided introducer placement, transducer leveling, and waveform identification. Additional content addressed recognition and management of waveform artifacts including underdamping, overdamping, and overwedging.

#### Simulation component

The simulation component provided structured, hands-on training using a hybrid model integrating physical task trainers with virtual hemodynamic monitoring. Each fellow paired with a critical care nurse educator and attending physician had the opportunity to perform essential steps of RHC including transducer leveling, catheter flushing, balloon inflation and deflation, and end-expiratory PAWP measurement (Figure S1). Participants determined CO using TD and iFick methods and calculated PVR. Training emphasized differentiation of normal and abnormal TD curves and incorporated acceptance or rejection of TD waveforms to reinforce appropriate injectate delivery technique (Figure S2). Fellows were encouraged to repeat simulation components as needed to support skill acquisition.

A procedural checklist (Supplemental) was piloted during the program. The checklist was informed by review of RHC templates from 7 institutions and finalized in collaboration with the cardiac catheterization laboratory director to ensure alignment with current guideline recommendations.^1^^,^^31^^,^^32^ The checklist prompted emphasis on essential hemodynamic parameters including right atrial and ventricular pressures; systolic, diastolic, and mean pulmonary artery pressures; PAWP; heart rate; CO by TD and iFick; PVR; and systemic and pulmonary blood gases for assessment of oxygen gradients and shunt physiology. To maximize participation, the program was delivered twice to account for fellows’ clinical schedules.

## Results

Of the 141 individuals invited to participate, 85 responded to the survey, yielding a response rate of 60.3%. After excluding 2 incomplete responses, 83 surveys were included in the final analysis. The distribution of survey respondents is summarized in Table 2. Nurses were trained in critical care but were not assigned to a specific specialty.

**Table 2:** Survey respondents by specialty.

	Total *(n = *83)	Pulmonary critical care	Cardiology
**Attending physician**	27	20	7
**Fellow physician**	18	11	7
**Critical care nurse**	38	N/A	

Analysis of survey responses stratified by provider type identified several knowledge areas meeting or exceeding the 70% performance threshold (highlighted in blue Table S3A). Zero-reference level confirmation was routinely performed by 77.8% of attending physicians and 72.2% of fellows. Measurement of PAWP at end-expiration was reported by 70.4% of attendings and 71.1% of nurses. Vasodilator challenge was incorporated by 70.4% of attending physicians. Additionally, the internal jugular vein was the preferred access site for RHC selected by 74.1% of attendings and 72.2% of fellows.

No single method for measuring CO—dFick, iFick, or TD—was dominant. Notably, TD and iFick were rarely used in combination. Pulmonary arterial wedge pressure was reported as obtained “always” by 34.9% and “often” by 22.9% of respondents. A mean of 3 PAWP measurements was obtained “always” by 6.0% and “often” by 18.1%. Confirmatory oxygen saturation in the wedge position was obtained in only 36.1% of cases across all provider types. Esophageal pressure monitoring in obese patients (body mass index > 30 kg/m^2^) was reported as “never” or “rarely” used by 85.5% of respondents.

Documentation of hemodynamic waveforms occurred in 65% of cases, as printed waveforms (42.1%), and/or screenshots uploaded to the electronic medical record (22.9%). In contrast, 45.8% of cases lacked waveform documentation. Fluid challenge was reported as “always” part of the standard RHC procedure in 8.4% of cases and “never” in 15.7%. The most common approach to TD CO measurement involved taking the best of 3 rapid bolus injections (55.4%).

A comparative analysis of responses from attendings and fellows by specialty (PCCM vs Cardiology) (Table S3B), revealed several areas meeting the 70% performance threshold. These included zero-reference level confirmation with nursing (reported by PCCM and cardiology attendings and PCCM fellows), PAWO_2_ measurement to confirm wedge position (cardiology attendings), PAWP measurement at end-expiration (PCCM attendings and ­cardiology fellows), routine use of vasodilator challenge (PCCM attendings and cardiology fellows), and preference for internal jugular vein as the access site (PCCM and cardiology attendings and PCCM fellows).

Knowledge gaps and opportunities for improvement include differentiating dFick vs iFick methods for CO measurement, performing TD CO assessments, accurately measuring PAWP at end-expiration, and obtaining and averaging multiple PAWP measurements.

### Educational intervention

One of the original objectives was to deliver the training program to attendings, fellows, and nurses in a unified interprofessional format. Logistical constraints made this impractical. Limitations included insufficient space to accommodate a large group, potential disruption to patient care by removing multiple providers from clinical duties, and the added burden on clinical colleagues covering service obligations. Additionally, institutional differences in policies regarding conference scheduling and attendance outside regular work hours further complicated implementation.

Given strong interest among fellows in additional RHC training, the educational intervention was primarily targeted to this group. Critical care nurse educators, along with attending physicians from PCCM and cardiology, attended the didactic portion of the program to preserve the interprofessional collaboration and teamwork essential for RHC performance. During the simulation sessions, each fellow was paired with a critical care nurse educator and attending physician.

Survey findings informed the design of the blended learning platform^36^ program for PCCM and cardiology fellows. Key teaching points extracted from survey data included identification of the zero-reference level,^6^^,^^34^ confirmation of wedge position using PAWO_2_,^3^^,^^31^ measurement of PAWP at end-expiration (Figure S1),^6^ differentiation between dFick and iFick methods (Table S2),^6^ and troubleshooting TD-derived CO tracings (Figure S2)^6^^,^^38^

A total of 11 fellows (5 PCCM, 6 cardiology; postgraduate year 1-3) completed the program.

### Pre- and posttraining outcomes

Survey data demonstrated notable improvements in several key areas (Table 3):

**Table 3:** Fellow pre- and postblended learning right heart catheterization program responses.

	Pretraining	Posttraining
**Direct Fick^a^**	22.3%	0.0%
**Indirect Fick^a^**	22.2%	81.8%
**Thermodilution (TD)^b^**	11.1%	9.1%
**Indirect Fick and thermodilution^a^**	0%	9.1%
**Zero-reference level**	72.2%	80.8%
**Obtain wedge**	38.9%	81.8%
**Wedge end-expiration**	66.7%	45.5%
**Number of TD “shoots”^a^**	50.0%	63.6%

a Includes always and often responses.

b Includes always responses.

- Identification of iFick as most common CO method: increased from 22.2% to 81.8%
- Zero-reference level confirmation by proceduralist with nurse: increased from 72.2% to 80.8%
- Consistent acquisition of wedge pressure: increased from 38.9% to 81.8%
- Obtaining best 3 TD rapid bolus injections: increased from 50.0% to 63.6%
- Use of both iFick and TD for CO: increased from 0.0% to 9.1%.

Conversely, correct identification of end-expiratory wedge pressure declined posttraining (66.7%-45.5%), highlighting the need for greater emphasis on this skill in future sessions.

## Discussion

The evolution of RHC training reflects broader changes in procedural education and the growing complexity of hemodynamic assessment. Innovations by Swan and Ganz to the original Nobel Prize-winning right heart catheter introduced the balloon-tipped and thermodilution technique for CO measurement, further advancing the clinical utility of RHC.^39-41^

Historically, RHC training followed an apprenticeship model, with learners acquiring skills through observation and supervised practice. Didactic instruction in physiology was often disconnected from hands-on experience. Ideally, a contemporary educational strategy should incorporate simulation-based training, competency-based assessment, interprofessional collaboration and teamwork, and digital learning modalities (Supplement, Right Heart Catheterization Training Program). Importantly, RHC is now taught across multiple disciplines including PCCM, cardiology, anesthesiology, and advanced practice nursing.

Accurate interpretation of RHC hemodynamic data remains essential.^31^^,^^32^ Our survey revealed widespread misconceptions among clinicians regarding CO measurement methods, a key parameter for calculating PVR and classifying pulmonary hypertension.^1^^,^^3^ Many respondents incorrectly believed that dFick is commonly used, despite its infrequent application in clinical practice due to technical challenges of measuring oxygen consumption at bedside.^1^^,^^3^ This confusion may be compounded by catheterization reports that list Fick CO without specifying whether the measurement was derived via direct or indirect methods. Our training program therefore included a comparative review of the 3 primary CO measurement techniques: TD, dFick, and iFick (Table S2). Posttraining assessments demonstrated improved knowledge of CO methodologies. While dFick remains the gold standard according to the Seventh World Symposium on Pulmonary Hypertension,^1^ its use in routine clinical practice is rare.

Accurate measurement of PAWP is equally critical. Posttraining data revealed persistent variability in identifying end-expiration and averaging wedge pressures, likely reflecting inconsistent practices during fellowship training and broader heterogeneity in clinical approaches. National surveys corroborate this finding, reporting that only 42% of clinicians routinely confirm wedge position using oxygen saturation and up to 30% remain uncertain about which CO method to trust when discrepancies occur.^42^^,^^43^ These observations underscore the need for standardized RHC protocols and consistent waveform documentation, particularly when the interpreting physician is not the proceduralist.^26-28^

Our prior experience with interprofessional education initiatives^44-46^ underscores the importance of team-based care in managing pulmonary hypertension and PAH. Participation in high-performing interprofessional pulmonary hypertension team meetings—comprising pulmonologists, cardiologists, advanced practice providers, social workers, and pharmacists—enhances fellows’ understanding of collaborative decision making. Although recent ACGME revisions have deemphasized procedural performance of RHC for PCCM fellows, competency in hemodynamic interpretation remains essential. Per ACGME requirements, cardiology fellows must demonstrate proficiency in performing RHC safely, interpreting results, and understanding procedural risks and indications.^37^ Fellows should also engage meaningfully in interprofessional discussions and anticipate expected hemodynamic findings based on preprocedural evaluations. These skills are particularly critical in community settings, where patients may be geographically distant from tertiary pulmonary hypertension centers. Ultimately, invasive RHC hemodynamic data must be interpreted within the broader clinical context to ensure accurate pulmonary hypertension classification and optimal patient care.

### Limitations

This project has several limitations. It was conducted at a single center and relied on survey data introducing potential recall bias as participants may have inaccurately reported prior experiences based on current beliefs or practices. The sustainability and scalability of the educational intervention depend on faculty availability and protected time. Additionally, while multiple-choice assessments provided insight into knowledge acquisition, they may not fully translate to procedural competence. Future efforts should incorporate performance-based evaluations and real-time feedback mechanisms. In the current educational climate, the importance of structured, simulation-based preparation prior to performing procedures on patients is appreciated.

Finally, the study was not powered for statistical testing. As a quality improvement and educational initiative, the focus was more exploratory in nature and on absolute improvements in knowledge. Any statistical testing would have a large probability of type I or type II errors given the sample size.

### Future directions

To our knowledge, this is the first reported blended platform learning program^36^ to comprehensively address the measurement and interpretation of CO, PAWP, and PVR through a combination of didactic instruction and hands-on simulation. The curriculum was designed to incorporate multimodal strategies while emphasizing practical engagement with RHC equipment and hemodynamic data. Although nurse-specific sessions were conducted separately under the direction of nursing leadership, future research should compare the effectiveness of interprofessional vs discipline-specific training formats.

Persistent challenges in RHC education include variability in procedural exposure and supervision; inconsistent emphasis on waveform interpretation; and advanced techniques such as vasodilator challenge, fluid challenge, esophageal monitoring, and limited formal assessment of procedural competence across training programs. A forward-looking innovative curriculum should integrate simulation-based procedural training, structured curricula with defined milestones, interprofessional education and team-building exercises, digital tools and case-based learning modules, direct observation with formative feedback, and competency-based assessment strategies. Greater context is given to each of these elements in our accompanying Right Heart Catheterization Training Program curriculum (Supplement). This curriculum provides a comprehensive framework for improving knowledge, technical proficiency, and interpretive skills essential for high-quality RHC performance in pulmonary hypertension and PAH.

## Conclusion

This study highlights critical opportunities for improving knowledge and aligning RHC procedural practice with current guidelines for pulmonary hypertension and PAH.^1^^,^^3^ Significant opportunities for learning were identified regarding the measurement of CO and PAWP—both essential for accurate hemodynamic assessment. These findings underscore the impact of diverse training backgrounds and institutional practices on procedural consistency. To ensure high-quality, evidence-based care, it is imperative to uphold procedural standards aligned with current guidelines. Ongoing interprofessional education emphasizing collaboration and periodic reassessment of team knowledge are essential to keep pace with evolving recommendations and optimizing outcomes for patients with pulmonary hypertension and PAH.

Portions of this manuscript were presented in abstract form at CHEST Annual Meeting 2023 (Honolulu, Hawaii).

## Supplementary Material

aapag022_Supplementary_Data
