# Supplementary material for: Right heart catheterization blended training program: quality improvement and educational outcomes
Source: ATS Sch. 2026 Apr 28;7(3):192–200. doi: 10.1093/atsscholar/aapag022 (PMC13519314; doi:10.1093/atsscholar/aapag022)
Supplement: Supplementary file 1 [file aapag022_supplementary_data.zip › aapag022_Supplementary_Data/IMPROVING_RIGHT_HEART_CATHETERIZATION_TECHNIQUE_QUESTIONNAIRE_12132025.pdf]

IMPROVING RIGHT HEART CATHETERIZATION TECHNIQUE QUESTIONNAIRE

Supplemental material for FARMER & ASGHAR, 2026

Dear survey responder:

To continue to improve the quality of right heart catheterization (RHC) to diagnose pulmonary hypertension, we are conducting a baseline survey of current practice. We appreciate your time to complete this short anonymous questionnaire. There are no right or wrong answers. Your responses will help us address any gaps in order to standardize our work to keep with current guidelines and improve patient care and outcomes.

1. Are you a: (Select one best answer)

___Critical care intensivist attending

___Pulmonary critical care attending

___Critical care fellow

___ Pulmonary critical care fellow

___Critical care nurse

___Interventional cardiologist

___Cardiologist

___Cardiology fellow

___Heart and vascular advanced practitioner

___Other, specify ____________________

2. Where do you perform right heart catheterizations? (Select all that apply)

___ Cardiac catheterization laboratory

___ MICU

___ CCU/HVCC

___SICU

___Other location, specify: _____________________

3. What type of Swan Ganz catheter do you use for right heart catheterization? (Check all that apply)
   a. Edward’s Life Science 110 cm 8 French thermodilution catheter

b. Edward’s Life Science 90 cm 5 French bipolar pacing catheter
c. I don’t know
d. Others specify ________

4. What do you like about this type of Swan Ganz catheter? (Check all that apply)
   a. Easy to use
   b. Only one available
   c. Only one I am trained to use
   d. Other: _______________

5. Is there another Swan Ganz you have used somewhere else that you wish we had here? Y/N
   a. If so, what was the make and model? (free text)

6. What is your preferred site of access when performing right heart catheterization?
   a. Femoral vein
   b. Internal jugular vein
   c. Brachial vein
   d. Other
   e. Not applicable

7. How do you confirm the accuracy of the hemodynamic data during the right heart catheterization process?

a. Provider checks the zero-reference level (also known as phlebostatic axis) with the nurse
b. Only Nurse checks the zero-reference level (phlebostatic axis)
c. I don’t know
d. Other

8. How often do you

a. Calculate cardiac output/cardiac Index (CO/CI) using the following method?

-Direct Fick	Never – Rarely – Sometimes – Often - Always
-Indirect Fick	Never – Rarely – Sometimes – Often - Always
-Thermodilution	Never – Rarely – Sometimes – Often - Always

b. Obtain a wedge? Never – Rarely – Sometimes – Often – Always

c. Calculate the mean of 3 wedge pressures? Never – Rarely – Sometimes – Often –Always

9. How often do patients undergo a fluid challenge during right heart catheterization?

Never --- Rarely --- Sometimes --- Often --- Always

10. When calculating thermodilution cardiac output, how many bolus injections are typically performed?
    a. 2
    b. 3
    c. 4
    d. 5 or more
    e. No typical number

11. Is confirmatory oxygen saturation obtained with right heart catheter in the wedge position?
    a. Yes
    b. No

12. Is esophageal probe used in patients with BMI => 30 kg/m^2?
    e. Yes
    f. No

13. Are cardiac output and wedge routinely obtained during exercise?
    g. Yes
    h. No

14. Do you currently use fluoroscopy during right heart catheterization?
    a. Yes
    b. No

14. If not, would you like it to be available?

a. Yes

b. No

15. Under what circumstances do you perform a vasodilator challenge? (Check all that apply)

    a. Suspect idiopathic pulmonary arterial hypertension

    b. Suspect heritable pulmonary arterial hypertension

    c. Suspect drug and toxin induced pulmonary arterial hypertension

    d. Vasodilator challenge is not included as part of right heart catheterization procedure

    e. Other: please specify

16. What type of vasodilator do you use for this challenge? (check all that apply)

    a. inhaled nitric oxide

    b. inhaled iloprost

    c. intravenous adenosine

    d. intravenous epoprostenol

    e. Another agent, specify_______

17. How are waveforms documented during RHC procedure? (Check all that apply)

    a. Waveforms are printed out from the monitor during right heart catheter insertion

    b. A screenshot of waveforms on the monitor is uploaded immediately to EMR

    c. Hemodynamic numbers are recorded and uploaded to EMR without waveforms

    d. Waveforms are not included in the documentation or report for this procedure

    e. Other, specify________________

18. At what point in the respiratory cycle is the wedge pressure obtained?

    a. Beginning of inspiration

    b. End of inspiration

    c. Beginning of expiration

    d. End of expiration

19. Which of the following is an ABSOLUTE contraindication to right heart catheterization?

    a. Arrhythmia

    b. Right-sided heart thrombus

c. Anemia

d. Coagulopathy

20. How familiar are you with the Hemosphere system for display of CO/CI?

a. Very familiar, use it regularly

b. Somewhat familiar, use it sometimes

c. Heard of it, no training to use it

d. Never heard of it, do not use it

21. Please add any comment that you think would be helpful for us to know.

22. Which of the following correctly describes the location of the phlebostatic axis?

a. 5th intercoastal space, level of ventricle, midaxillary line

b. 4th intercoastal space, level of atria, posterior axillary line

c. 5th intercoastal space, level of ventricle, anterior axillary line

d. 4th intercostal space, level of atria, midaxillary line
