# Supplementary material for: Right heart catheterization blended training program: quality improvement and educational outcomes
Source: ATS Sch. 2026 Apr 28;7(3):192–200. doi: 10.1093/atsscholar/aapag022 (PMC13519314; doi:10.1093/atsscholar/aapag022)
Supplement: Supplementary file 1 [file aapag022_supplementary_data.zip › aapag022_Supplementary_Data/PROCEDURAL_CHECKLIST_FOR_RIGHT_HEART_CATHETERIZATION_V2.pdf]

RIGHT HEART CATHETERIZATION (Pulmonary artery catheterization)

Supplemental information for FARMER & ASGHAR, 2026

Checklist for Procedural Fellows

I. Pre-Procedure Preparation

☐ Confirm indication for right heart catheterization
☐ Obtain **written informed consent** (or from health care proxy if patient unable)
☐ Perform **hand hygiene**
☐ Apply **maximal sterile barrier precautions**
– Sterile gown, gloves, hat
– Mask with face shield or eye protection
– Full body sterile drape

☐ Position patient in **Trendelenburg** for introducer insertion (unless contraindicated)

II. Equipment and Setup

☐ 9 Fr introducer
☐ 7.5 Fr Swan-Ganz catheter
☐ Pressure tubing and transducer setup
☐ Line cart with sterile supplies

☐ Check pressure bag pressure
☐ Level transducer zero port to **phlebostatic axis (right atrium)**
– 4th intercostal space, mid-anterior/posterior chest
☐ Re-level transducer after **any patient position change**
☐ Zero transducer to **atmospheric pressure**

III. Introducer Insertion

☐ Identify insertion site using **bedside ultrasound**
☐ Perform **chlorhexidine skin prep**
– 30 seconds back-and-forth scrub
– Allow **2 minutes dry time**
☐ Infiltrate site with **1% lidocaine**
☐ Insert introducer using sterile technique
☐ Aspirate blood from side port
☐ Flush side port with normal saline

IV. Catheter Preparation

☐ Place sterile sleeve over catheter **before balloon testing**
☐ Inflate balloon **once** with **1.5 mL air**
⚠️ Do **not** use saline or water
☐ Inspect balloon for leaks and symmetry

☐ Connect pressure tubing to catheter
– PA distal port = **yellow**
– CVP proximal port = **blue**

☐ Prime and flush **all ports**
– PA distal (yellow): exits catheter tip
– CVP proximal (blue): exits below 30 cm mark
– VIP/white port: exits above 30 cm mark

☐ Shake catheter tip to assess waveform fidelity
☐ If waveform dampened: flush and reassess

☐ Identify catheter length markings
– Small lines = 10 cm
– Large lines = 50 cm

V. Catheter Insertion and Advancement

☐ Select PA scale on monitor (e.g., **40 mmHg scale**)
☐ Insert catheter to **20 cm**, then **inflate balloon**
☐ Advance catheter with **balloon inflated** (float forward)
☐ **Deflate balloon when withdrawing**

☐ Continuously observe pressure waveforms during advancement

VI. Waveform Identification and Hemodynamics

☐ Identify **Right Atrial (RA) waveform**
– Measure RAP (normal **1–6 mmHg**)

☐ Identify **Right Ventricular (RV) waveform**
– Measure RV pressure
– Normal: **15–25 / 1–8 mmHg**

☐ Identify **Pulmonary Artery (PA) waveform**
– Measure PA systolic/diastolic/mean
– Normal: **15–25 / 4–12 / ~14 mmHg**

☐ Identify **Pulmonary Artery Wedge (PAWP) waveform**
– Normal: **4–12 mmHg**
– <18 mmHg after fluid challenge

VII. Cardiac Output and Oxygenation

☐ Measure cardiac output
– **Thermodilution preferred** unless direct Fick available
☐ If needed, calculate via **indirect Fick**

☐ Measure oxygen saturations
– Mixed venous (SvO_2)
– Pulmonary artery O_2
– Arterial O_2 (SaO_2)

☐ Measure systemic blood pressure

VIII. Calculations

☐ Pulmonary vascular resistance (PVR)
– (mPAP – PAWP) / CO
– Normal **≤ 2 Wood units**

☐ Systemic vascular resistance (SVR)
– (MAP – RAP) / CO
– Normal **800–1200 dynes·sec/cm^5** (10–15 WU)

☐ Cardiac index
– CO / BSA
– Normal **2.4–4.0 L/min/m^2**

☐ Transpulmonary gradient
– mPAP – PAWP (normal ≤12 mmHg)

☐ Diastolic pressure gradient
– Diastolic PAP – PAWP (normal <6 mmHg)

IX. Completion and Post-Procedure

☐ Transfer waveforms to medical record
☐ Determine need for **vasodilator** or **fluid challenge**
☐ Withdraw PA catheter
☐ Remove introducer
☐ Apply **manual pressure** and confirm hemostasis
