# Supplementary material for: Right heart catheterization blended training program: quality improvement and educational outcomes
Source: ATS Sch. 2026 Apr 28;7(3):192–200. doi: 10.1093/atsscholar/aapag022 (PMC13519314; doi:10.1093/atsscholar/aapag022)
Supplement: Supplementary file 1 [file aapag022_supplementary_data.zip › aapag022_Supplementary_Data/TABLE S1S2S3.pdf]

TABLE S1

Hemodynamic criteria of pulmonary hypertension (PH) [4]

**Category**	**Hemodynamic Characteristics**
PH	mPAP > 20 mmHg
Pre-capillary PH	mPAP > 20 mmHg<br>PAWP ≤ 15 mmHg<br>PVR > 2 WU
Isolated post-capillary PH (ipcPH)	mPAP > 20 mmHg<br>PAWP > 15 mmHg<br>PVR ≤ 2 WU
Combined post- and pre-capillary PH (cpcPH)	mPAP > 20 mmHg<br>PAWP > 15 mmHg<br>PVR > 2 WU
Exercise PH	mPAP/CO slope > 3 mmHg/L/min between rest and exercise

LEGEND: mPAP: mean pulmonary arterial pressure; PAWP: pulmonary arterial wedge pressure; PVR: pulmonary vascular resistance; WU: Wood Units; CO: cardiac output

TABLE S2 Comparison of methods to determine cardiac output [6]

**Method**	**Principle**	**Procedure**	**Advantages**	**Disadvantages**
**Thermodilution**	Based on temperature changes in blood after injecting a cold saline solution	Cold saline is injected into the right atrium, and temperature change is measured in the pulmonary artery	Provides continuous monitoring, useful in critical care settings<br>Clinical GOLD standard	Affected by intracardiac shunts, tricuspid regurgitation, and low cardiac output states
**Direct Fick**	Uses the ratio of oxygen consumption to the difference in arterial and venous oxygen content	Directly measures oxygen consumption (VO2) and arterial-venous oxygen difference	Accurate and reliable in stable conditions	Difficult to measure VO2, especially in critically ill or obese patients<br>Labor intensive<br>More widely used in research
**Indirect Fick**	Estimates oxygen consumption using pulse oximetry instead of directly measuring mixed venous oxygen saturation	Uses estimated VO2 instead of directly measured VO2	Easier to perform than direct Fick, less invasive	Less accurate than direct Fick, affected by estimation errors

TABLE S3A	SUMMARY OF SURVEY RESPONSES			
**Survey question Possible answer**	**Responses**			
	**Attending**	**Fellow**	**Nurse**	**Total**
	**N= 27 (32.5%)**	**N=18 (21.7%)**	**N=38 (45.8%)**	**N=83**
**Zero reference level (ZRL)**				
Proceduralist & nurse check ZRL	21 (77.8%)	13 (72.2%)	17 (44.7%)	51 (61.4%)
Nurse checks ZRL	5 (18.5%)	6 (33.3%)	14 (36.8%)	25 (30.1%)
I don't know	0 (0.0%)	0 (0.0%)	5 (13.2%)	5 (6.0%)
Other*	0 (0.0%)	0 (0.0%)	2 (5.3%)	2 (2.4%)
**Confirmatory wedge oxygen saturation**				
No	13 (48.1%)	10 (55.6%)	24 (63.2%)	47 (56.6%)
Yes	12 (44.4%)	7 (38.9%)	11 (28.9%)	30 (36.1%)
**Cardiac output method**				
Direct Fick				
Never	4 (14.8%)	3 (16.7%)	18 (47.4%)	25 (30.1%)
Rarely	9 (33.3%)	4 (22.2%)	5 (13.2%)	18 (21.7%)
Sometimes	4 (14.8%)	4 (22.2%)	3 (7.9%)	11 (13.3%)
Often	3 (11.1%)	3 (16.7%)	4 (10.5%)	10 (12.0%)
Always	3 (11.1%)	1 (5.6%)	5 (13.2%)	9 (10.8%)
Indirect Fick				
Never	6 (25.0%)	3 (16.7%)	13 (34.2%)	22 (26.5%)
Rarely	10(37.0%)	3 (16.7%)	8 (21.1%)	21 (25.3%)
Sometimes	5 (18.5%)	5 (27.8%)	1 (2.6%)	11 (13.3%)
Often	3 (11.1%)	2 (11.1%)	5 (13.2%)	10 (12.0%)
Always	0 (0.0%)	2 (11.1%)	7 (18.4%)	9 (10.8%)
Thermodilution				
Never	5 (18.5%)	3 (16.7%)	8 (21.1%)	16 (19.3%)
Rarely	4 (14.8%)	5 (27.8%)	6 (15.8%)	15 (18.1%)
Sometimes	5 (18.5%)	5 (27.8%)	7 (18.4%)	17 (20.5%)
Often	5 (18.5%)	1 (5.6%)	6 (15.8%)	12 (14.5%)
Always	3 (11.1%)	2 (11.1%)	8 (21.1%)	13 (15.7%)
Indirect Fick & Thermodilution				
Always	0 (0.0%)	0 (0.0%)	2 (5.3%)	2 (2.4%)
**Obtain wedge pressure**				
Never	1 (3.7%)	0 (0.0%)	1 (2.6%)	2 (2.4%)
Rarely	1 (3.7%)	2 (11.1%)	7 (18.4%)	10 (12.0%)
Sometimes	8 (29.6%)	3 (16.7%)	8 (21.1%)	19 (22.9%)
Often	7 (25.9%)	5 (27.8%)	7 (18.4%)	19 (22.9%)
Always	9 (33.3%)	7 (38.9%)	13 (34.2%)	29 (34.9%)
**Mean of 3 wedge pressures**				
Never	7 (25.9%)	3 (16.7%)	16 (42.1%)	26 (31.3%)
Rarely	10 (37.0%)	3 (16.7%)	6 (15.8%)	19 (22.9%)
Sometimes	5 (18.5%)	3 (16.7%)	3 (7.9%)	11 (13.3%)

Often	3 (11.1%)	5 (27.8%)	7 (18.4%)	15 (18.1%)
Always	0 (0.0%)	2 (11.1%)	3 (7.9%)	5 (6.0%)
**Measure wedge pressure**				
Beginning of inspiration	1 (3.7%)	2 (11.1%)	1 (2.6%)	4 (4.8%)
End of inspiration	5 (18.5%)	2 (11.1%)	4 (10.5%)	11 (13.3%)
Beginning of expiration	0 (0.0%)	1 (5.6%)	0 (0.0%)	1 (1.2%)
End of expiration	19 (70.4%)	12 (66.7%)	27 (71.1%)	58 (69.9%)
**Vasodilator challenge routinely performed**				
Disagree	8 (29.6%)	6 (33.3%)	14 (36.8%)	28 (33.7%)
Agree	19 (70.4%)	12 (66.7%)	26 (68.4%)	57 (68.7%)
**Preferred site of access for RHC**				
Femoral vein	0 (0.0%)	0 (0.0%)	0 (0.0%)	0 (0.0%)
Internal jugular vein	20 (74.1%)	13 (72.2%)	26 (68.4%)	59 (71.1%)
Brachial vein	2 (7.4%)	1 (5.6%)	3 (7.9%)	6 (7.2%)
Other	1 (3.7%)	2 (11.1%)	0 (0.0%)	3 (3.6%)
Not applicable	3 (11.1%)	2 (11.1%)	8 (21.1%)	13 (15.7%)
**Fluid challenge**				
Never	3(11.1%)	2(11.1%)	8(21.1%)	13(15,7%)
Rarely	8(29.6%)	5(27.8%)	12(31.6%)	25(30.1%)
Sometimes	10(37.0%)	9(50.0%)	12(31.6%)	31(37.3%)
Always	3(11.1%)	1(5.6%)	3(7.9%)	7(8.4%)
**Esophageal pressures**				
Never	16(59.3%)	12(66.7%)	21(55.3%)	49(59.0%)
Rarely	7(25.9%)	4(22.2%)	11(28.9%)	22(26.5%)
Sometimes	2(7.4%)	1(5.6%)	3(7.9%)	6(72%)
Often	0 (0.0%)	0 (0.0%)	0 (0.0%)	0 (0.0%)
Always	0 (0.0%)	0 (0.0%)	0 (0.0%)	0 (0.0%)
**Thermodilution number of shoots**				
Two	2(7.4%)	2(11.1%)	1(2.6%)	5(6.0%)
Three	14(51.9%)	9(50.0%)	23(60.5%)	46(55.4%)
Four	0 (0.0%)	0 (0.0%)	0 (0.0%)	0(0.0%)
Five or more	0 (0.0%)	0 (0.0%)	3(7.9%)	3(3.6%)
No particular number	8(29.6%)	6(33.3%)	8(21.1%)	22(26.5%)
**Hemodynamic waveform documentation (select all that apply)**				
Printed out during RHC and scanned to medical record	13 (48.1%)	9 (50.0%)	13 (34.2%)	35 (42.1%)
Screenshot from monitor uploaded to medical record	8 (29.6%)	4 (22.2%)	7 (18.4%)	19 (22.9%)
Hemodynamics without waveforms uploaded	13 (48.1%)	5 (27.8%)	20 (52.6%)	38 (45.8%)

TABLE S3B	SURVEY RESPONSES BY SPECIALTY			
**Survey question** **Possible answers**	**Responses**			
	**Attending PCCM**	**Attending Cardiology**	**Fellow PCCM**	**Fellow Cardiology**
	**N= 20**	**N=7**	**N=11**	**N=7**
**Zero reference level (ZRL)**				
Proceduralist & nurse check ZRL	16 (80.0%)	5 (71.4%)	9 (81.8%)	4 (57.1%)
Nurse checks ZRL	4 (20.0%)	1 (14.3%)	3 (27.3%)	3 (42.9%)
I don't know	0 (0.0%)	0 (0.0%)	0 (0.0%)	0 (0.0%)
Other*	0 (0.0%)	0 (0.0%)	0 (0.0%)	0 (0.0%)
**Confirmatory wedge oxygen saturation**				
No	12 (60.0%)	1 (14.3%)	5 (45.5%)	5(71.4%)
Yes	6 (30.0%)	6 (85.7%)	6 (54.5%)	1 (14.3%)
**Cardiac output method**				
Direct Fick				
Never	2 (10.0%)	2 (28.6%)	3 (27.3%)	0 (0.0%)
Rarely	7 (35.0%)	2 (28.6%)	3 (27.3%)	1 (14.3%)
Sometimes	4 (20.0%)	0 (0.0%)	3 (27.3%)	1 (14.3%)
Often	1 (5.0%)	2 (28.6%)	2 (18.2%)	1 (14.3%)
Always	2 (10.0%)	1 (14.3%)	0 (0.0%)	1 (14.3%)
Indirect Fick				
Never	4 (20.0%)	2 (28.6%)	3 (27.3%)	0 (0.0%)
Rarely	7 (35.0%)	3 (42.9%)	2 (18.2%)	1 (14.3%)
Sometimes	4 (20.0%)	1 (14.3%)	3 (27.3%)	2 (28.6%)
Often	2 (10.0%)	1 (14.3%)	2 (18.2%)	0 (0.0%)
Always	0 (0.0%)	0 (0.0%)	1 (9.1%)	1 (14.3%)
Thermodilution				
Never	3 (15.0%)	2 (28.6%)	1 (9.1%)	2 (28.6%)
Rarely	3 (15.0%)	1 (14.3%)	4 (36.4%)	1 (14.3%)
Sometimes	2 (10.0%)	3 (42.9%)	4 (36.4%)	1 (14.3%)
Often	5 (25.0%)	0 (0.0%)	1 (9.1%)	0 (0.0%)
Always	2 (10.0%)	1 (14.3%)	1 (9.1%)	1 (14.3%)
Indirect Fick & Thermodilution				
Always	0 (0.0%)	0 (0.0%)	0 (0.0%)	0 (0.0%)
**Obtain wedge pressure**				
Never	1 (5.0%)	0 (0.0%)	0 (0.0%)	0 (0.0%)
Rarely	0 (0.0%)	1 (14.3%)	1 (9.1%)	1 (14.3%)
Sometimes	7 (35.0%)	1 (14.3%)	3 (27.3%)	0 (0.0%)
Often	4 (20.0%)	3 (42.9%)	4 (36.4%)	1 (14.3%)
Always	7 (35.0%)	2 (28.6%)	3 (27.3%)	4 (57.1%)
**Mean of 3 wedge pressures**				
Never	5 (25.0%)	2 (28.6%)	2 (18.2%)	1 (14.3%)
Rarely	7 (35.0%)	3 (42.9%)	2 (18.2%)	1 (14.3%)
Sometimes	3 (15.0%)	2 (28.6%)	2 (18.2%)	1 (14.3%)

Often	3 (15.0%)	0 (0.0%)	4 (36.4%)	1 (14.3%)
Always	0 (0.0%)	0 (0.0%)	1 (9.1%)	1 (14.3%)
**Measure wedge pressure**				
Beginning of inspiration	0 (0.0%)	1 (14.3%)	1 (9.1%)	1 (14.3%)
End of inspiration	3 (15.0%)	2 (28.6%)	2 (18.2%)	0 (0.0%)
Beginning of expiration	0 (0.0%)	0 (0.0%)	1 (9.1%)	0 (0.0%)
End of expiration	15 (75.0%)	4 (57.1%)	7 (63.6%)	5 (71.4%)
**Vasodilator challenge routinely performed**				
Disagree	4 (20.0%)	4 (57.1%)	4 (36.4%)	2 (28.6%)
Agree	16 (80.0%)	3 (42.9%)	7 (63.6%)	5 (71.4%)
**Preferred site of access for RHC**				
Femoral vein	0 (0.0%)	0 (0.0%)	0 (0.0%)	0 (0.0%)
Internal jugular vein	15 (75.0%)	5 (71.4%)	10 (90.9%)	3 (42.9%)
Brachial vein	1 (5.0%)	1 (14.3%)	0 (0.0%)	1 (14.3%)
Other	1 (5.0%)	0 (0.0%)	0 (0.0%)	2 (28.6%)
Not applicable	2 (10.0%)	1 (14.3%)	1 (9.1%)	1 (14.3%)
**Fluid challenge**				
Never	2 (10.0%)	1 (14.3%)	1 (9.1%)	1 (14.3%)
Rarely	6 (30.0%)	2 (28.6%)	3 (27.3%)	2 (28.6%)
Sometimes	6 (30.0%)	4 (57.1%)	7 (63.6%)	2 (28.6%)
Always	3 (15.0%)	0 (0.0%)	0 (0.0%)	1 (14.3%)
**Esophageal pressures**				
Never	12 (60.0%)	4 (57.1%)	6 (54.5%)	6 (85.7%)
Rarely	4 (20.0%)	3 (42.9%)	4 (36.4%)	0 (0.0%)
Sometimes	2 (10.0%)	0 (0.0%)	1 (9.1%)	0 (0.0%)
Often	0 (0.0%)	0 (0.0%)	0 (0.0%)	0 (0.0%)
Always	0 (0.0%)	0 (0.0%)	0 (0.0%)	0 (0.0%)
**Thermodilution number of shoots**				
Two	2 (10.0%)	0 (0.0%)	2 (18.2%)	0 (0.0%)
Three	10 (50.0%)	4 (57.1%)	5 (45.5%)	4 (57.1%)
Four	0 (0.0%)	0 (0.0%)	0 (0.0%)	0 (0.0%)
Five or more	0 (0.0%)	0 (0.0%)	0 (0.0%)	0 (0.0%)
No particular number	5 (25.0%)	3 (42.9%)	4 (36.4%)	2 (28.6%)
**Hemodynamic waveform documentation (select all that apply)**				
Printed out during RHC and scanned to medical record	11 (55.0%)	2 (28.6%)	7 (63.6%)	2 (28.6%)
Screenshot from monitor uploaded to medical record	7 (35.0%)	1 (14.3%)	2 (18.2%)	2 (28.6%)

Hemodynamics without waveforms uploaded	10 (50.0%)	3 (42.9%)	3 (27.3%)	2 (28.6%)
